# Stage-Specific Role of Interferon-Gamma in Experimental Autoimmune Encephalomyelitis and Multiple Sclerosis

**DOI:** 10.3389/fimmu.2015.00492

**Published:** 2015-09-29

**Authors:** Gabriel Arellano, Payton A. Ottum, Lilian I. Reyes, Paula I. Burgos, Rodrigo Naves

**Affiliations:** ^1^Immunology Program, Biomedical Sciences Institute, School of Medicine, Universidad de Chile, Santiago, Chile; ^2^Faculty of Science, Universidad San Sebastián, Santiago, Chile; ^3^Department of Clinical Immunology and Rheumatology, School of Medicine, Pontificia Universidad Católica de Chile, Santiago, Chile

**Keywords:** interferon-gamma, experimental autoimmune encephalomyelitis, multiple sclerosis, innate immunity, adaptive immunity, neuroinflammation

## Abstract

The role of interferon (IFN)-γ in multiple sclerosis (MS) and its animal model, experimental autoimmune encephalomyelitis (EAE), has remained as an enigmatic paradox for more than 30 years. Several studies attribute this cytokine a prominent proinflammatory and pathogenic function in these pathologies. However, accumulating evidence shows that IFN-γ also plays a protective role inducing regulatory cell activity and modulating the effector T cell response. Several innate and adaptive immune cells also develop opposite functions strongly associated with the production of IFN-γ in EAE. Even the suppressive activity of different types of regulatory cells is dependent on IFN-γ. Interestingly, recent data supports a stage-specific participation of IFN-γ in EAE providing a plausible explanation for previous conflicting results. In this review, we will summarize and discuss such literature, emphasizing the protective role of IFN-γ on immune cells. These findings are fundamental to understand the complex role of IFN-γ in the pathogenesis of these diseases and can provide basis for potential stage-specific therapy for MS targeting IFN-γ-signaling or IFN-γ-producing immune cells.

## Introduction

Interferon (IFN)-γ is the only type II IFN family member. It is secreted by activated immune cells, mainly T and natural killer (NK) cells, but also B cells, NKT cells, and professional antigen presenting cells (APC). IFN-γ binds to a heterodimeric receptor, IFNGR, expressed ubiquitously on almost all cell types. Given its pleiotropic functions, IFN-γ plays a pivotal role in orchestrating immune system homeostasis (1–4). Historically, IFN-γ production has been considered the hallmark of T helper (Th)1 cells driving inflammation and autoimmunity, such as multiple sclerosis (MS). MS is an inflammatory and demyelinating disorder of the central nervous system (CNS) and is the leading cause of non-traumatic neurological disability in young adults (5). According to the clinical course, MS can be classified in different types: relapsing-remitting disease (RRMS), consisting of acute recurrent attacks followed by a variable degree of recovery, and progressive forms characterized by chronic and irreversible neurological disability (6).

To date, experimental autoimmune encephalomyelitis (EAE) remains as the animal model most widely used to study the immunopathological mechanisms and therapeutic approaches to MS (7, 8). EAE is induced by immunization with myelin-derived antigens in adjuvant or by the adoptive transfer of activated myelin-specific T cells into syngeneic naive hosts. First, an initiation/inductive phase occurs, where innate and adaptive immune cells are antigen stimulated in the periphery. That is followed by the effector phase characterized by an acute immune cell infiltration into the CNS, and a later chronic phase of inflammation and axonal damage (9).

Discrepant results have been reported in relation to the role of IFN-γ in EAE and MS (3, 4, 10). Factors such as dose, site specificity, and timing of action as well as interaction with other cytokines and cells can determine the net effect of IFN-γ (3, 10, Ottum et al., in preparation). Recent evidence supports a, not mutually exclusive, stage-specific role of IFN-γ in EAE providing an explanation to these controversial results and a model whereby this cytokine can both promote and limit the development of these pathologies. In this same Research Topic, we have reviewed the opposing roles of IFN-γ on CNS-resident cells in EAE and MS (Ottum et al., in preparation). Here, we will review the evidence on IFN-γ’s dual role in the cells of the immune system in these same pathologies.

## Two-Faced Role of IFN-γ in EAE and MS

Initially, a positive association between increased levels of IFN-γ and demyelinating lesions in the CNS in MS and EAE attributed this cytokine a pathological role (11–15). In mice, passive immunization of healthy animals with encephalitogenic Th1 lymphocytes producing IFN-γ was sufficiently capable of inducing EAE (16). Besides, mice deficient in T-bet, a transcription factor that drives Th1 differentiation, were protected from developing EAE (17, 18). The proinflammatory effects of IFN-γ were confirmed in a pilot clinical study showing that seven of eighteen RRMS patients treated with IFN-γ exhibited symptom exacerbations (19). Consistently, secondary progressive MS patients (SPMS) treated with antibodies against IFN-γ exhibited slightly reduced clinical symptoms (20).

However, subsequent studies have challenged the notion that IFN-γ is pathogenic, and there is accumulating evidence proposing a protective role for IFN-γ in EAE and MS. Systemic or intraventricular injection of IFN-γ in EAE mice reduced the severity of disease symptoms, morbidity, and mortality (21, 22), and systemic IFN-γ treatment in chronic-relapsing EAE (CREAE) significantly delayed the appearance of relapses (23). Likewise, anti-IFN-γ therapy exacerbated EAE symptoms and made a mice strain resistant to EAE susceptible to developing disease (21–26). These results have been corroborated using animals deficient in the IFN-γ gene, which showed increased incidence of EAE, earlier disease onset and more severe symptoms compared with control mice (27–29). Even more, animals lacking IFNGR developed EAE with higher susceptibility, severity, and lethality (30–32). Passive transfer of encephalitogenic splenic cells from EAE-induced IFNGR-deficient mice into either wild-type (WT) or IFNGR-deficient recipient mice led to the development of EAE, but only WT mice recovered from illness (33). Interestingly, in tumor necrosis factor (TNF)-α receptor-deficient mice, a higher frequency of Th1 cells and enhanced mRNA expression of IFN-γ in the CNS was associated with a milder EAE (34).

Finally, in the marmoset EAE model, administration of human IFN-γ did not aggravate clinical symptoms, and by contrast, there was a trend to delay the appearance of the neurological episodes associated with less inflammation and demyelination during the EAE late phase (35). Regarding MS, induction of endogenous IFNs production in progressive MS patients showed that some patients with improving symptoms had high levels of serum ­IFN-γ, while clinical worsening was related with low serum ­IFN-γ levels (36).

## Stage-Specific Role of IFN-γ

The opposing activities that IFN-γ has in MS and the different models of EAE remain unresolved. However, collective evidence has shown that these paradoxical functions likely reflect a disease stage-specific opposing role of IFN-γ in EAE: promoting pathogenesis during the initiation phase but immunosuppression in the effector phase. Delivery of an intrathecal IFN-γ expression system during the initiation phase triggered an earlier disease onset followed by recovery, while overexpression of IFN-γ in the chronic phase resulted in disease amelioration (37). Consistently, intraventricular injection of IFN-γ during the initiation phase in CREAE mice increased the number of relapses (38). More recently, Naves et al. showed that IFNGR-deficient mice exhibited delayed disease onset followed by a more severe chronic phase, compared to WT mice (31). Similar results have been found analyzing mice lacking the IFN-γ gene or injecting an anti-IFN-γ neutralizing antibody during EAE progression (39). Furthermore, the administration of IFN-γ to EAE mice during the inductive period led to disease exacerbation, while such treatment was protective during the effector phase (31). Interestingly, the immunosuppressive activity of IFN-γ required functional type I IFN signaling and signal transducer and activator of transcription (STAT)-1 (31). In this way, stage-specific functions of IFN-γ can reconcile previous conflicting results in EAE and might also explain the mixed outcome reported in RRMS patients treated with IFN-γ (19).

## IFN-γ and Immune Cells

**Figure 1:**
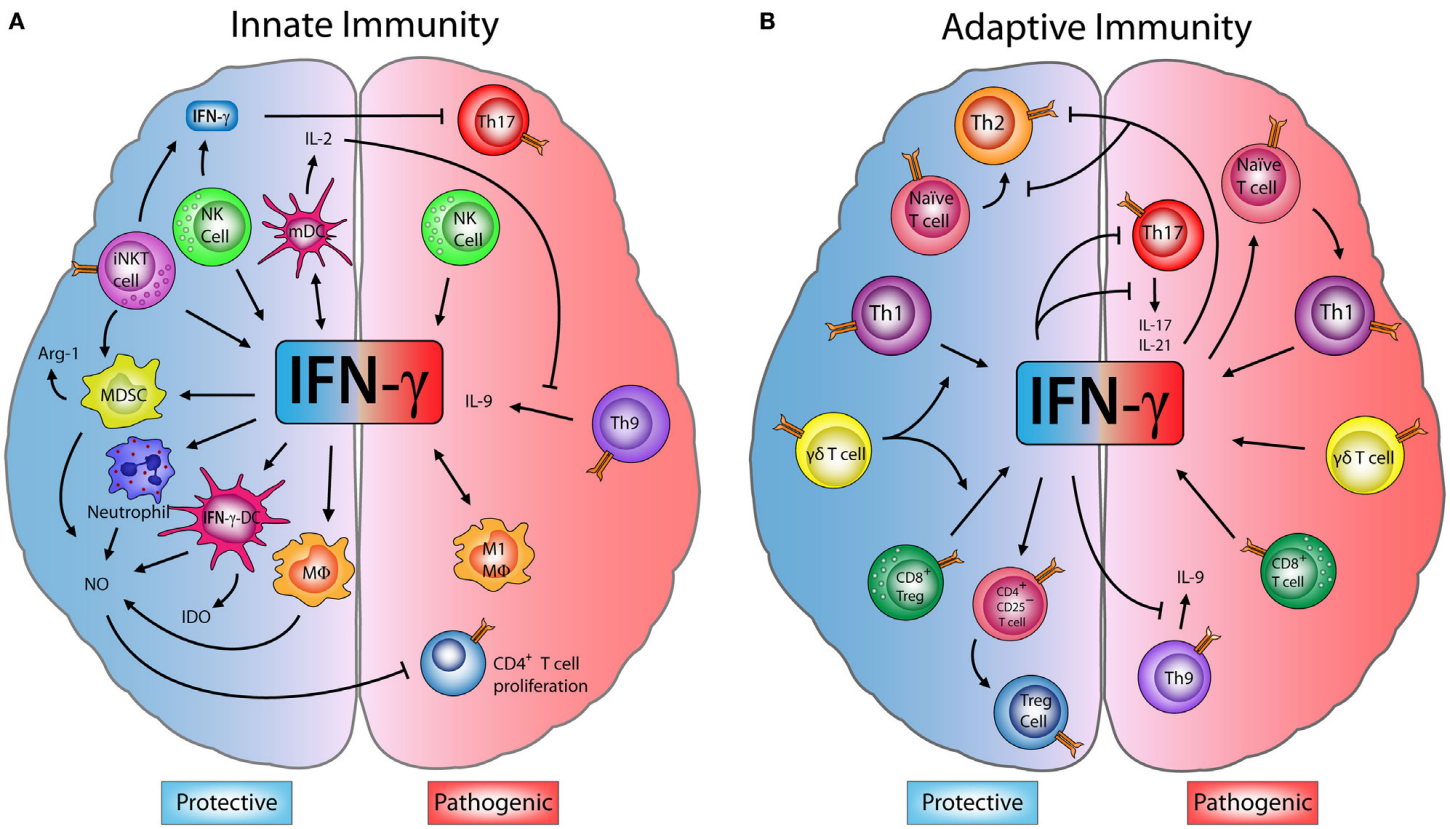
**Dual role of IFN-**γ** in innate and adaptive immune cells in EAE**. **(A)** *Innate immunity*: M1-macrophages (M1-MΦ) and natural killer (NK) cells produce interferon (IFN)-γ, which has a pathogenic role exacerbating encephalomyelitis autoimmune experimental (EAE) symptoms. However, some studies have shown that IFN-γ produced by NK and invariant NKT (iNKT) cells inhibits effector Th17 cells, decreasing the disease severity. IFN-γ induces the production of nitric oxide (NO) in neutrophils, MΦ, myeloid-derived suppressor cells (MDSCs), and IFN-γ-induced dendritic cells (IFN-γ-DC). NO can directly inhibit the proliferation of CD4^+^ T cells. IFN-γ also induces the expression of indoleamine 2,3-dioxygenase (IDO) in IFN-γ-DC and arginase-1 (Arg-1) by MDSC, enzymes that can suppress inflammation. Furthermore, IFN-γ induces IL-27 production by mature dendritic cells (mDC) which blocks Th9 differentiation and IL-9 production, controlling disease progression. **(B)** *Adaptive immunity*: IFN-γ secreted by Th1 and CD8^+^ T cells has an inflammatory effect and can drive the onset and progression of EAE. Despite this, IFN-γ is able to block Th9 cells, while Th1-secreted IFN-γ inhibits Th2 and Th17 effector cells. Interestingly, IFN-γ can induce CD4^+^CD25^+^ regulatory T cells (Tregs) increasing their FOXP3 expression. Upon transfer, these IFN-γ-induced Tregs limit the severity of EAE. Moreover, IFN-γ production by CD8^+^ regulatory T cells (CD8^+^ Tregs) also reduces EAE symptoms. Finally, IFN-γ produced by γδ T cells worsens EAE, but has a regulatory role on the production of IFN-γ by T cells, which is necessary to limit disease.

Compelling evidence shows that IFN-γ exerts opposing effects on immune cells during the development of EAE and MS. In addition, several innate and adaptive immune cells play a dual role during the progression of these diseases associated with their IFN-γ production (Figure 1). Below, we will review and discuss this literature, focusing on the less-known protective face of IFN-γ (Table 1).

**Table 1:** **The protective effects of IFN-**γ** and IFN-**γ**-producing immune cells in EAE and MS**.

Cell type	Experimental design	Effects of IFN-**γ**	Reference
Macrophages	*In vitro* culture of IFNGR-deficient PEC	IFN-γ induces PEC NO-expression inhibiting proliferation of splenocytes	(33)
Neutrophils	Induced EAE in IFN-γ and IFNGR-deficient mice	IFN-γ restricts neutrophils infiltration in the brainstem and cerebellum primarily by regulating CXCL2 expression	(40–42)
	*In vitro* analysis of Gr1^+^ neutrophils sorted from CNS of mice with EAE	IFN-γ secreted by T cells induced NO production by Gr1^+^ neutrophils which inhibited T cell proliferation	(43)
Myeloid-derived suppressor cells (MDSCs)	Analysis of CD11b^+^ Gr1^+^ MDSC from EAE mice	IFN-γ secreted by activated T cells induced MDSC inhibiting CD4^+^ T cells proliferation by NO-dependent manner	(44)
	EAE mice treated with anti-IFN-γ	Anti-IFN-γ reduced MDSCs frequency and increased EAE severity	(45)
Natural killer cells (NK)	EAE mice treated with anti-IFN-γ	Decreased Th17-characteristic transcription factors expression due to modulation of microglia activation	(46)
	HINT1/Hsp70 protein complex from brains of PLP-sensitized SJL/J mice injected into congenic mice before immunization	Upregulated MHC class I peptide H60 expression, increased NK cell IFN-γ production, inhibited IL-17 production, and prevented EAE	(47–49)
	Analysis of NK cell functionality in human PBMC	RRMS patients exhibit impaired response to IL-12 and severely diminished IFN-γ production in CD3^−^CD56^bright^CD16^−^ NK cells	(50)
Invariant NKT cells	*In vivo* IFN-γ neutralization in αGalCer-treated mice with EAE. *In vitro* iNKT analysis	Increased production of IFN-γ, IL-4, and IL-10 by iNKT cells which mediated the suppression of Th17 cells and increased EAE regulation by MDSCs	(51–53)
Dendritic cells (DC)	Transfer of IFN-γ treated DC into murine EAE models	Induced an incompletely mature DC phenotype and decreased disease severity and relapse frequency	(54)
	*In vitro* analysis of splenocytes isolated from WT and IFN-γ-deficient EAE mice	Induced DC IL-27 expression which inhibited Th9 cell differentiation and IL-9 production by Th9 and Th17 cells	(55)
CD4^+^ T lymphocytes	IFN-γ added to CD3-activated PBMC from chronic-progressive MS patients	Lymphocyte proliferation inhibition in an IFN-γ dose-dependent manner	(56)
	Analysis of IFN-γ deficient mice with EAE	Increased apoptosis and inhibited proliferation *in vivo* and *ex vivo* of CD4^+^CD44^high^ T cells in spleen and CNS	(57)
	Study of IFN-γ and IFNGR EAE deficient mice	Inhibited Th17 differentiation and IL-17 production	(31, 58–62)
	IFN-γ deficient EAE mice treated with anti-IL-9	Decreased Th9 differentiation and IL-9 production *in vitro* and *in vivo* in the CNS of mice with EAE	(55)
	CD4^+^ T cells transfected with IFN-γ expressing vector transferred into EAE mice	Th1 IFN-γ^high^CD25^−^FOXP3^−^ suppresses Th17 effector cells and decreased EAE severity	(63)
γδ T cells	EAE generated in bone marrow chimera with γδ and IFN-γ-deficient mice	γδ T cells promotes the expression of IFN-γ by T cells with a reduction of EAE severity	(64)
CD4^+^ Tregs	*In vitro* addition of IFN-γ to mice and human CD4^+^CD25^−^ T cell cultures. IFN-γ-converted Tregs injected into EAE mice	IFN-γ-converted Tregs inhibited T cell proliferation in mice and human cells. Administration of these cells ameliorated EAE severity	(65)
CD8^+^ T lymphocytes	Transfer of MOG-induced CD8^+^ T cells from IFN-γ-deficient mice into wild-type mice before EAE induction	Amelioration of EAE severity mediated by CD8^+^ T cell IFN-γ production	(66)
	Analysis of CD8^+^LAP^+^ T cells from IFN-γ and IFNGR-deficient mice and transfer into EAE	IFN-γ production by CD8^+^LAP^+^ T cells inhibited T cell proliferation and reduced severity of EAE.	(67)
	Vaccination with a TCR-derived peptide before EAE induction in WT and IFN-γ KO mice	Vaccination activates CD8αα^+^TCRαβ^+^ T cells and delayed EAE onset in an IFN-γ mediated fashion	(68–70)
	Isolation of human and mice CD8^+^CD38^high^ T cells. *In vivo* injection of CD8^+^CD38^high^ into EAE mice	IFN-γ production by CD8^+^CD38^high^ T cells inhibit T cell proliferation in human and mice. These cells decreased disease severity and delayed onset of EAE.	(71)
	MS patients and EAE mice treated with Glatiramer acetate (GA)	GA increases CD8^+^ T proliferation and IFN-γ levels in MS and IDO and IFN-γ-producing CD8^+^ T cells in EAE	(72, 73)
B cells	IFN-γ treatment in early EAE stage in marmoset	Reduced plasma MOG-specific IgG levels	(35)

### Innate immune cells

#### Macrophages and Neutrophils

IFN-γ controls the infiltration of macrophages and neutrophils into the CNS regulating the course of EAE (74). Animals deficient in IFN-γ or IFNGR generate an atypical disease affecting mainly the brainstem and cerebellum with increased expression of CXCL2, favoring the recruitment of high numbers of CXCR2-mediated neutrophils; while in conventional EAE, IFN-γ leads to increased CCL2 levels guiding macrophage infiltration into the spinal cord mediated by CCR2 (40–42). Macrophages and neutrophils produce high levels of nitric oxide (NO), which has both pathogenic and regulatory functions in neuroinflammation (75). Interestingly, IFN-γ is a primary inducer of NO and mice deficient in inducible nitric oxide synthase (iNOS) develop a severe form of EAE (76, 77). Willenborg et al. showed that peritoneal exudate cells (PEC), characterized by a high presence of macrophages, are able to inhibit the extensive proliferation of splenocytes from IFNGR-deficient mice with EAE by IFN-γ-dependent NO production (33). Neutrophils and myeloid-derived suppressor cells (MDSCs) with high expression of Gr-1 also exhibited potent suppressor activity in EAE, inhibiting T cell proliferation through a mechanism that was absolutely dependent on IFN-γ and NO (43–45). Additionally, IFN-γ along with interleukin (IL)-4, secreted by activated invariant NKT (iNKT) cells, stimulated MDSCs to suppress EAE via iNOS and arginase (arg)-1 expression (51).

#### Natural Killer Cells

NK cells play both a regulatory and pathogenic role in EAE and MS (39, 46, 78–86). Although the underlying mechanisms are poorly understood, several studies suggest that IFN-γ-producing NK cells might be driving this duality in a location and stage-dependent manner (39, 46–49, 87). NK cells have been identified as the main source of IFN-γ production in the initiation stage of EAE, which might be necessary for migration of pathogenic T cells into the CNS (39). Interestingly, early but not late depletion of NK cells significantly delayed the onset of disease (39). IL-18 and IL-21 are two key cytokines involved in NK cell functional maturation (88, 89). Administration of IL-21 before EAE immunization promoted higher IFN-γ production by NK cells and induced a significantly enhanced acute phase with more intense CNS cell infiltration compared to untreated mice (90). However, IL-21 treatment failed to induce augmentation of IFN-γ production and had no effect on disease progression when applied for one week starting a few days before disease onset (90). Similarly, IL-18 injection in WT mice at the time of immunization enhanced disease severity promoting autoreactive Th1 cell development through the induction of IFN-γ by NK cells (87). Moreover, IFN-γ signaling in NK cells was required to restore EAE susceptibility in IL-18 defective mice (87). Taken together, these results suggest that early IFN-γ production by NK cells mainly contributes to the initiation, but not progression, of EAE pathogenesis. By contrast, once NK cells infiltrate the CNS, they assume a protective role suppressing myelin-reactive Th17 cells via modulation of microglia activation. This effect was CNS compartment-restricted and was perforin and IFN-γ-dependent (46). Therefore, signals and/or components generated in the CNS during the effector phase might be inducing protective functions in infiltrating NK cells. Indeed, peptides complexed with the chaperone heat shock protein (Hsp) 70 derived from inflamed brain of EAE mice have been described as promotors of the immunotolerogenic activity of NK cells in EAE. Suppressive effects of Hsp-peptide complex-activated NK cells correlated with high production of IFN-γ and resulted in inhibition of Th17 cells (47–49).

NK cell subtypes and iNKT cells may also have IFN-γ-mediated suppressive activity in MS and EAE. Regarding MS, evidence in two independent cohorts of RRMS patients demonstrated that the classically inhibitory CD3^−^CD56^bright^CD16^−^ NK cells from RRMS patients have impaired expansion in response to IL-12, and severely diminished IFN-γ production compared to healthy control NK cells (50). In addition, *in vivo* activation of iNKT cells at the same time as EAE induction significantly ameliorated disease progression through mechanisms dependent on IFN-γ alone (52) or synergistically with IL-4 and IL-10, resulting in inhibition of the Th17 response (53).

#### Dendritic Cells

Dendritic cells (DC) are professional APC important to maintain the balance between immunity and tolerance. In EAE, they efficiently present myelin antigens in order to prime and polarize naïve T cells. They also help regulate EAE severity as evidenced by disease exacerbation in DC deficient mice (91, 92). The regulatory effects of DC are partly due to the IFN-γ-induced production of IL-27 that suppressed the differentiation and encephalitogenicity of Th9 cells. It also inhibited the production of IL-9 by both Th9 and Th17 cells. This suppression was partially dependent on STAT-1 and T-bet and was necessary to regulate EAE severity (55). Remarkably, splenic DC exposed to IFN-γ for 48 hours exhibited an immature and tolerogenic phenotype (tol-DC). These tol-DC decreased disease severity in Lewis rats and relapse frequency in SJL/J and B6 mouse models when transferred during the inductive phase (54). EAE amelioration was accompanied by reduced macrophage activation and CD4^+^ T cell CNS infiltration, compared to control mice. The therapeutic activity was dependent on an antigen-specific IFN-γ pathway, involving increased DC expression of indoleamine 2,3-dioxygenase (IDO), which induced CD4^+^ T cell apoptosis (54).

### Adaptive immune cells

#### CD4^+^ T Lymphocytes

CD4^+^ T (Th) cells proliferate and differentiate into various subtypes in response to antigen stimulation and their microenvironment in order to exert specific effector or regulatory functions (93). Effector CD4^+^ T (Teff) cell lineages, such as Th1 cells, Th2 cells, Th17 cells, and Th9 cells, and regulatory T cells (Tregs) can be distinguished by the cytokines they produce and the transcription factors essential for their differentiation. These T cells also exhibit functional and phenotypical plasticity expressing cytokines and/or transcription factors of other lineages (94, 95).

Classically, IFN-γ is known for promoting the differentiation of Th1 cells and inhibiting the Th2 immune response which may contribute to neuroinflammation (5, 10, 96, 97). Despite its inflammatory activity, IFN-γ increased apoptosis and inhibited proliferation of CD4^+^CD44^high^ (activated) T lymphocytes from both the spleen and CNS of EAE mice (57). Notably, it also inhibited *in vitro* proliferation of T cell receptor (TCR)-activated peripheral blood mononuclear cells (PBMC) from progressive MS patients in a dose-dependent manner (56). Mice depleted of IFN-γ or IFN-γ signaling developed more severe EAE, atypical neurological symptoms, and increased Th17-characteristic inflammation. These data underscore an important anti-inflammatory function of IFN-γ in EAE: the inhibition of pathogenic Th17 cell differentiation and cytokine production (5, 31, 58–62, 96). Besides, it has been shown that IFN-γ has a STAT-1-mediated direct inhibitory effect on pathogenic Th9 cells (55). Interestingly, another study identified a non-pathogenic Th1 cell subset with high IFN-γ expression, capable of restraining EAE development during early stages of disease by suppressing Th17 cells in an IFN-γ-dependent manner (63). The inhibitory mechanism involved the activation of STAT-1 and IL-21 expression via induction of T-bet (60, 62). Despite the ability of IFN-γ to directly and indirectly inhibit Th17 cells, a pathogenic population of Th1 cells has been identified in EAE and MS that also expresses IL-17. This capacity to express both cytokines (IFN-γ and IL-17) may be due to the plasticity of Th17 cells, which can undergo a shift toward the Th1 phenotype (95, 98, 99).

#### γδ T Cells

Several studies have shown that γδ T cells are present in the CNS of MS patients and EAE mice (100). Given that activated γδ T cells have the capacity to produce high expression of Th1 and Th17 cytokines, they might contribute to the induction or maintenance of neuroinflammation. However, efforts to determine a role for these cells have given contradictory results. While some studies have found that depletion of γδ T cells resulted in reduced severity of EAE, other reports have described disease aggravation (100). Regarding IFN-γ, evidence suggests that during early EAE, γδ T cells may act either as a main source of this cytokine (101) or regulate IFN-γ expression in other cell types, including CD4^+^ and CD8^+^ T cells (64). Indeed, SJL/J mice depleted of γδ T cells showed a significant reduction of IFN-γ expression in the CNS at all stages of EAE (102). Other studies have shown that mice deficient in γδ T cells that are reconstituted with γδ T cells lacking IFN-γ expression developed a significantly delayed and attenuated EAE. This suggested that IFN-γ production by γδ T cells may be central to initial inflammatory events (101). Despite this, Ponomarev et al. proposed that γδ T cells are required to promote CNS-restricted production of sufficient levels of IFN-γ necessary for EAE recovery (64).

#### CD4^+^ Regulatory T Lymphocytes

It has been reported that IFN-γ is important to the function of Tregs in EAE and MS. Reduced FoxP3 expression and lower frequency and function of Tregs was reported in IFN-γ-deficient mice with EAE, in comparison to EAE-induced WT mice (65). Remarkably, treatment of CD4^+^CD25^−^ T cells from WT or IFN-γ-deficient mice with IFN-γ alone or with additional TCR stimulation led to their conversion into Tregs expressing CD25 and FOXP3 (65). These IFN-γ-induced Tregs effectively inhibited EAE disease progression when adoptively transferred into IFN-γ-deficient mice. Human CD4^+^CD25^−^ T cells from healthy volunteers were similarly converted into functionally active Tregs *ex vivo* upon IFN-γ stimulation (65).

A new subpopulation of Tregs expressing T-bet, CXCR3 and IFN-γ, named Th1-like Tregs has been reported in healthy individuals (103) and have regulatory functions focused on Th1-mediated inflammatory diseases (104–108). Interestingly, these cells were also described in MS and EAE (109, 110). An increased frequency of Th1-like Tregs with reduced suppressive function was reported in untreated RRMS patients compared to healthy controls (110). In this case, addition of IFN-γ neutralizing antibodies recovered their functionality suggesting that IFN-γ might contribute to their reduced immunomodulatory capacity (110).

#### CD8^+^ T Lymphocytes

Several studies have demonstrated that IFN-γ production by CD8^+^ T cells is a major mediator of EAE induced by cytotoxic T lymphocytes (CTL) (111–114). One of these investigations showed that atypical EAE induced by intrathecal transfer of myelin basic protein (MBP)-specific CD8^+^ T cells in C3H mice was ameliorated by co-injection with neutralizing antibodies for IFN-γ (112). Other studies have identified subsets of regulatory CD8^+^ T cells (CD8^+^ Tregs) that suppress EAE development via IFN-γ-dependent mechanisms. Both therapeutic and prophylactic transfer of myelin oligodendroctye glycoprotein MOG-induced CD8^+^ T cells into mice with EAE ameliorated disease suppressing the chronic phase, but not affecting the disease onset or acute phase (66, 115). Strikingly, this protective function was lost when IFN-γ-deficient MOG-induced-CD8^+^ T cells were transferred before EAE induction in WT mice, but was enhanced when IFN-γ production was stimulated in MOG-specific CD8^+^ T cells before cell transfer (66). Furthermore, in those studies reporting a pathogenic function, myelin-specific CD8^+^ T cell lines used to passively induce EAE were generated from CD8^+^ T cells isolated during the inductive phase (111, 112). In contrast, regulatory myelin-specific CD8^+^ T cells were obtained during the chronic phase of disease (66, 115). Taken together, these results reinforce the notion of a stage-specific IFN-γ-dependent regulation, mediated in this case by CNS-specific regulatory CD8^+^ T cells.

A naturally occurring CD8^+^ Tregs subset was identified that expressed latency-associated peptide (LAP) on their cell surface and produced more IFN-γ than their LAP^−^ counterparts. Adoptive transfer of these cells previous to myelin immunization improved EAE recovery mediated by their IFN-γ production (67). A CD8αα^+^TCRαβ^+^ T cell subset capable of preventing EAE when stimulated with a TCR-derived peptide before MBP-peptide immunization in H-2u mouse strains has also been described (68, 69, 116). Interestingly, the vaccine failed to prevent EAE development in IFN-γ-deficient mice and resulted in delayed disease onset but worsened disease severity compared to control mice, suggesting an important stage-specific role for IFN-γ signaling in CD8αα^+^TCRαβ^+^ T cell-mediated protection (70, 116). A CD8^+^ Tregs subtype expressing high levels of CD38 ectonucleotidase suppressed Teff cell proliferation in a non-antigen specific, cell-to-cell contact, and IFN-γ-dependent fashion, resulting in ameliorated EAE (71). Finally, IFN-γ-producing CD8^+^ T cells induced by glatiramer acetate (GA), a therapy for MS, suppressed EAE in mice via an IDO-dependent mechanism, suggesting that the immunomodulatory action of GA is mediated at least in part by IFN-γ production by CD8^+^ T cells (72). Consistently, GA-specific CD8^+^ T cells from GA-treated RRMS patients tended to produce more IFN-γ than CD8^+^ T cells from untreated patients (73). In progressive MS patients, CTL had impaired IL-2 induced IFN-γ production and decreased ability to suppress proliferation of TCR-stimulated autologous lymphocytes (56).

#### B Lymphocytes

The effect of IFN-γ on B cells in the neuroinflammatory context of MS and EAE is unclear. Bar-Or and colleagues demonstrated that CD19^+^ B cells isolated from RRMS patients had significantly increased production of lymphotoxin (TNF-β) and TNF-α in response to IFN-γ and insignificant changes in IL-10 production (117). In marmoset EAE, exogenous administration of IFN-γ caused no significant clinical change in disease; however, there was a significant decrease in plasma IgG specific to MOG peptides (35).

## Concluding Remarks

Recent studies support the notion that IFN-γ exerts a stage-specific role during EAE development. Strikingly, several innate and adaptive immune cells develop opposite activities during EAE progression, which is related to their production of IFN-γ in a stage-specific manner. Furthermore, the suppressive activity of different types of immune regulatory cells is IFN-γ-dependent. Taken together, these data provide a mechanistic basis explaining the previous controversial results in relation to the role of IFN-γ in EAE and MS. Delineating the varying activities of IFN-γ as well as the role of IFN-γ-producing immune cells during the course of EAE and MS will not only provide insight into the complex role of IFN-γ in these diseases but might also lead to therapies targeting IFN-γ signaling or IFN-γ-producing immune cells. These treatments can be helpful to a selective group of MS patients or during a specific stage of disease.

## Conflict of Interest Statement

The authors declare that the research was conducted in the absence of any commercial or financial relationships that could be construed as a potential conflict of interest.

## Funding

This review was supported by DIUSS N° 2012-0004-R (LIR), FONDECYT 1140049 (RN) and 1141211 (PIB), and the CONICYT Doctoral fellowship 21130452 (GA).
